# Japanese encephalitis vaccine-specific envelope protein E138K mutation does not attenuate virulence of West Nile virus

**DOI:** 10.1038/s41541-019-0146-0

**Published:** 2019-12-05

**Authors:** Jaclyn A. Kaiser, Huanle Luo, Steven G. Widen, Thomas G. Wood, Claire Y-H. Huang, Tian Wang, Alan D. T. Barrett

**Affiliations:** 1 Department of Microbiology and Immunology, University of Texas Medical Branch, Galveston, TX 77555 USA; 2 Department of Biochemistry and Molecular Biology, University of Texas Medical Branch, Galveston, TX 77555 USA; 3 Division of Vector-Borne Diseases, Centers for Disease Control and Prevention, Fort Collins, CO 80521 USA; 4 Department of Pathology, University of Texas Medical Branch, Galveston, TX 77555 USA; 5 Sealy Institute for Vaccine Sciences, University of Texas Medical Branch, Galveston, TX 77555 USA

**Keywords:** Live attenuated vaccines

## Abstract

West Nile (WNV) and Japanese encephalitis viruses (JEV) are closely related, mosquito-borne neurotropic flaviviruses. Although there are no licensed human vaccines for WNV, JEV has multiple human vaccines, including the live, attenuated vaccine SA14-14-2. Investigations into determinants of attenuation of JE SA14-14-2 demonstrated that envelope (E) protein mutation E138K was crucial to the attenuation of mouse virulence. As WNV is closely related to JEV, we investigated whether or not the E-E138K mutation would be beneficial to be included in a candidate live attenuated WNV vaccine. Rather than conferring a mouse attenuated phenotype, the WNV E-E138K mutant reverted and retained a wild-type mouse virulence phenotype. Next-generation sequencing analysis demonstrated that, although the consensus sequence of the mutant had the E-E138K mutation, there was increased variation in the E protein, including a single-nucleotide variant (SNV) revertant to the wild-type glutamic acid residue. Modeling of the E protein and analysis of SNVs showed that reversion was likely due to the inability of critical E-protein residues to be compatible electrostatically. Therefore, this mutation may not be reliable for inclusion in candidate live attenuated vaccines in related flaviviruses, such as WNV, and care must be taken in translation of attenuating mutations from one virus to another virus, even if they are closely related.

## West Nile Virus: live vaccine attenuation

Japanese encephalitis virus (JEV) and West Nile virus (WNV) are closely related neurotropic viruses—a live attenuated vaccine exists for JEV but not for WNV. A team led by Alan D.T. Barrett at the University of Texas investigated whether a key E-protein mutation (E138K) in the live attenuated JEV vaccine can also attenuate a candidate live WNV vaccine. The mutant WNV vaccine shows essentially identical behavior to the virulent parental strain in vitro but unexpectedly also has unimpaired lethality and neurotropism when mice are challenged intraperitoneally. Sequencing of the mutant WNV vaccine demonstrated increased frequencies of single-nucleotide variants clustered around residue 138—including reversion to the parental strain glutamic acid. E138K mutation is therefore unlikely to be a reliable means to attenuate candidate WNV vaccines.

## Introduction

West Nile virus (WNV) is a mosquito-borne member of the flavivirus genus, which has a single-stranded, positive-sense RNA genome of ~11 kilobases that encodes a single polyprotein.^1^ The polyprotein is co- and posttranslationally cleaved into three structural proteins (capsid, membrane, and envelope) and seven non-structural (NS) proteins (NS1, NS2A, NS2B, NS3, NS4A, NS4B, and NS5). Although there are many mosquito-borne flaviviruses of public health significance including dengue (DENV), yellow fever (YFV), and Zika viruses transmitted by *Aedes aegypti*, WNV clusters serologically and phylogenetically with Japanese encephalitis virus (JEV).^1^ WNV and JEV are both neurotropic flaviviruses that can cause potentially fatal disease in humans and are transmitted by *Culex spp.* mosquitoes. Between 2002 and 2018, the United States Centers for Disease Control and Prevention received reports ranging from 386 to 2946 cases of West Nile-induced neuroinvasive disease (WNND) annually as well as from 32 to 286 annual fatalities.^2,3^ In addition, 2018 had the largest recorded outbreak of WNV in Europe with >2000 reported cases.^4^ WNND can manifest as encephalitis, meningitis, and acute flaccid paralysis, and infected individuals can have long-lasting neurological sequelae with ~10% of WNND cases being fatal.^1^

Importantly, there are multiple live attenuated and inactivated vaccines licensed for use in humans to prevent disease from JEV;^5^ however, there are no licensed human vaccines to prevent WNV disease. The most widely used JEV vaccine utilizes a live attenuated strain, SA14-14-2, which was derived empirically by serial passage of wild-type strain SA14 in primary hamster kidney cells.^6^ Multiple groups have investigated the genetic determinants of attenuation of SA14-14-2 and although there are vaccine-associated mutations spanning both the structural and NS protein genes of the virus, six mutations in the envelope (E) protein (L107F, E138K, I176V, T177A, Q264H, and K279M) were found to be indispensable to the attenuated phenotype.^7,8^

Of the SA14-14-2 vaccine-specific E-protein mutations, E138K stands out in the literature for its robust contribution to the attenuation of the mouse neuroinvasive and neurovirulent phenotype of JEV. Substitution of E138K in a wild-type JEV infectious clone strongly reduced the mouse neuroinvasive and neurovirulent phenotypes.^9^ A single site reversion of K138E in a JE SA14-14-2 infectious clone most profoundly increased neurovirulence compared with reversion of other vaccine-specific E-protein residues.^10^ Genomic sequencing of historical vaccine derivatives also supports that E138K is important for vaccine attenuation, as all attenuated derivatives of SA14-14-2 harbor this substitution.^11^ In sum, E-E138K seems to be critical for the attenuated phenotype of JE SA14-14-2. A sequence alignment of the E genes of wild-type strains of JEV and WNV showed that the glutamic acid residue at 138 is conserved between JEV and WNV strains. The E-E138K mutation has been investigated in cell culture-adapted WNV, an infectious clone based on a lineage 2 WNV isolate, and a chimeric WNV/YFV 17D virus, and the mutation was found to increase attenuation to different degrees in each virus backbone.^12–15^ As E-E138K had not previously been studied by rational mutation of the virulent lineage 1 NY99 infectious clone (NY99ic), the phenotype of this mutant was investigated in detail. It was hypothesized that the E-E138K mutation in WNV NY99ic would attenuate mouse neuroinvasion and neurovirulence, and be beneficial for inclusion in a candidate live attenuated WNV vaccine; however, this was not found to be correct. Rather, the virulent mouse phenotype of WNV NY99ic was retained. Analysis of E-protein structural modeling and single-nucleotide variants (SNVs) provided a plausible explanation for the reversion to wild-type phenotype.

## Results

### Recovery of the E-E138K Mutant

The WNV E-E138K mutant was recovered from transfected Vero cells with the engineered E-E138K mutation plus a single synonymous C1077U nucleotide mutation from NY99ic, but no additional mutations were present in the consensus sequence of the genome. The mutant multiplied to a high-infectivity titer comparable to that of NY99ic (>8 log_10_ plaque-forming units (PFU)/mL) and neither NY99ic nor the E-E138K mutant had a temperature-sensitive (TS) phenotype at 41 °C (data not shown).

### Multiplication Kinetics in Cell Culture

**Fig. 1:**
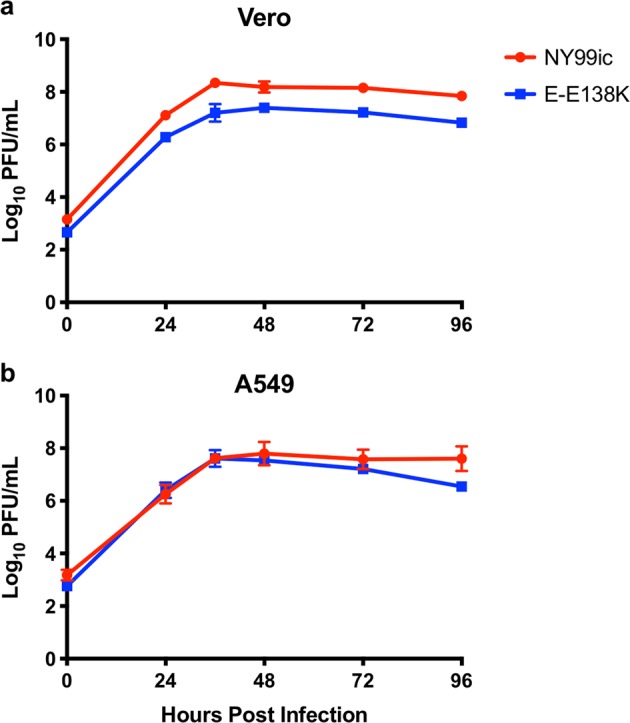
Multiplication kinetics of NY99ic and the E-E138K mutant are similar in Vero and A549 cells at a MOI of 0.1. Data points represent the mean viral titer and error bars represent the SD of two titrations of two biological replicates (four titrations in total). PFU plaque-forming units.

Multiplication kinetics in Vero and A549 cells were compared, to investigate differences in both interferon-I (IFN-I)-deficient and IFN-I-competent cell lines, respectively. In Vero cells, the E-E138K mutant multiplied to titers ~10-fold lower than NY99ic between 24 and 96 hours post infection (hpi); however, both viruses multiplied to high titers (Fig. 1a). In A549 cells, the two viruses had nearly identical multiplication kinetics, suggesting the E-E138K mutant does not have increased sensitivity to IFN-I at the time points measured (Fig. 1b).

### Induction of Cytokines and Chemokines in A549 Cells

**Fig. 2:**
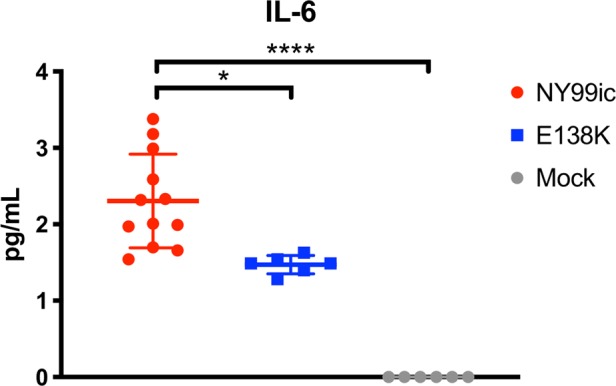
Extracellular IL-6 is lower in E-E138K-infected cells compared with NY99ic-infected cells. Cytokines were measured in A549 cell supernatant at 36 hpi. Error bars represent the SD for 12 (NY99ic) or 6 biological replicates (E-E138K and mock). *p = 0.03, ****p < 0.0001 in Kruskal–Wallis test with Dunn’s multiple comparisons. IL interleukin.

Supernatants from infected A549 cells isolated at 36 hpi were used to measure cytokines and chemokines. Seven cytokines in the multiplex were either undetectable or were only present at very low levels (<1 pg/mL) (Supplementary Table 1), which could be a limitation of cytokine signaling in A549 cells or of the specific time point investigated. Twenty-one cytokines, chemokines, and growth factors were not differentially induced between the two viruses and the only cytokine with a statistical difference was interleukin (IL)-6, for which the E-E138K mutant caused decreased secretion compared with NY99ic (*p* = 0.03 in Kruskal–Wallis test with Dunn’s multiple comparisons) (Fig. 2).

### Mouse Neuroinvasive Phenotype

The neuroinvasive phenotype of the WNV E-E138K mutant was compared with NY99ic in groups of five NIH Swiss Webster outbred mice inoculated by the intraperitoneal (i.p.) route. All mice inoculated with 500 PFU of NY99ic had a lethal infection and succumbed between 8 and 12 days post infection (dpi) (Table 1). Surprisingly, the 500 PFU inoculum of the E-E138K mutant was also completely lethal and all mice died between 7 and 11 dpi (Table 1). The experiment was repeated and the results were confirmed (10/10 mice succumbed in total). A further five mice inoculated with the high dose (82 million PFU) of the E-E138K mutant died more quickly (5–6 dpi) than the mice inoculated with 500 PFU (*p* = 0.009) (Table 1). In conclusion, the E-E138K mutation in WNV did not confer significant attenuation of mouse neuroinvasion, but instead it retained the virulent phenotype of NY99ic.

**Table 1:** The E-E138K mutant does not have an attenuated phenotype in vivo.

Virus (inoculum)	#Mice survived/total	Average survival time (days) ± SD
NY99ic (500 PFU)	0/10	9.0 ± 1.3
E138K (500 PFU)	0/10	9.4 ± 1.3
E138K (8.2 × 10^7^ PFU)	0/5	5.4 ± 0.5**

Groups of 4-week-old Swiss Webster outbred mice were inoculated by the intraperitoneal (i.p.) route. *PFU* plaque forming unit

Significance was tested using a Kruskal–Wallis test with Dunn’s multiple comparisons, to compare mutant survival time with NY99ic survival time

**p = 0.009

### Virus Isolation From Mouse Brain

Virus was collected from the brains of five mice that succumbed to the 500 PFU inoculum of the E-E138K mutant on either 8 or 10 dpi. One mouse that succumbed on 8 dpi had a mean virus titer in the brain of 8.7 log_10_ PFU/g and 4 mice that succumbed on 10 dpi had mean viral titers of 4.9, 5.6, 7.1, and 8.7 log_10_ PFU/g. Sequencing of the E gene of viral RNA from brain homogenate demonstrated that in all five mice the E-E138K mutation reverted to the wild-type glutamic acid residue.

### Next-Generation Sequencing Analysis

**Fig. 3:**
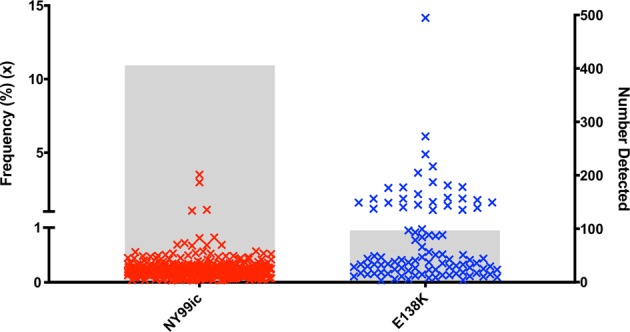
Single-nucleotide variant profiles of the unpassaged (P0) virus stocks differ between NY99ic and the E-E138K mutant. Each ‘x’ represents the frequency of a variant and the gray bars display the total number of variants detected.

**Fig. 4:**
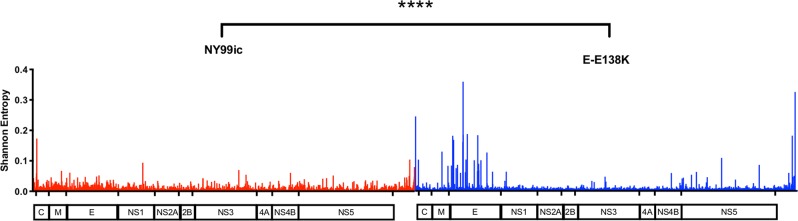
The E-E138K mutant has higher Shannon entropy across the genome than NY99ic. A Mann–Whitney test was used to compare the entropy of the entire genome for NY99ic and the E-E138K mutant. ****p < 0.0001.

To further investigate the stability of the WNV E-E138K mutant, the quasi-species diversity of the unpassaged (P0) cell culture stocks of NY99ic and the E-E138K mutant were compared using next-generation sequencing (NGS). Following deep sequencing of the entire genome, both NY99ic and the E-E138K mutant had a high depth of coverage averaging 6868 and 6983 reads, respectively. As the coverage was comparable between the two viruses, SNVs were measured without down sampling to a lower average coverage. Overall, 406 SNVs and length polymorphisms were detected in NY99ic, and they ranged in frequency from 0.03% to 3.51% (Fig. 3). Ninety-seven SNVs and length polymorphisms were detected in the E-E138K mutant quasi-species, and although there were fewer SNVs than in NY99ic, some SNVs in the E-E138K mutant population were present at a higher frequency (0.04%–14.16%). To investigate these differences in more detail, length polymorphisms were excluded and continued analysis was only undertaken on SNVs with a frequency of 1% or higher. This filtering reduced the number of SNVs in NY99ic to three subpopulations in the structural protein genes, whereas E-E138K had 26 subpopulations primarily located in the E-protein gene (Fig. 3 and Tables 2 and 3). The most prominent SNV in the E-E138K mutant population had a frequency of 14.2% and encoded a reversion of E-K138E (Table 3). There was no evidence of SNVs at E-138 in NY99ic. As an alternative measurement of diversity, Shannon entropy was calculated for NY99ic and the E-E138K mutant. The E-E138K mutant had more nucleotide diversity than NY99ic, and this was most evident in the E-protein gene (*p* < 0.0001) (Fig. 4). After a single passage (P1) and five passages (P5) in Vero cells, the E-E138K mutation was retained in the consensus sequence. Although both the P1 and P5 stocks of the E-E138K mutant had SNVs that encoded reversion at residue E-138, these SNVs had a significant strand bias according to Fisher’s exact tests (*p* = 0.0087 for P1, *p* = 6.95 × 10^−9^ for P5), and therefore they could be false positives.

**Table 2:** Summary of all single-nucleotide variants ≥1% of viral RNA populations of NY99ic in unpassaged (P0) virus stocks.

Nucleotide position	Major nucleotide	Minor nucleotide	Protein position	Major residue	Minor residue	Frequency (%)
120	C	G	C-8	P	P	3.5
831	A	G	prM-122	V	V	1.1
1072	A	G	E-36	K	E	1.1

**Table 3:** Summary of all single-nucleotide variants ≥1% of viral RNA populations of the E-E138K mutant in unpassaged (P0) virus stocks.

Nucleotide position	Major nucleotide	Minor nucleotide	Protein position	Major residue	Minor residue	Frequency (%)
767	A	U	prM-101	E	V	2.6
939	A	G	prM-158	L	L	1.5
1044	A	G	E-26	E	E	1.9
1077	U	C	E-37	D	D	3.6
1105	A	G	E-47	N	D	4.1
1216	A	G	E-84	K	E	1.6
1372	A	C	E-136	K	Q	3.0
1378	A	G	E-138	K	E	14.2
1380	G	A	E-138	K	K	6.1
1463	G	C	E-166	R	T	1.9
1464	A	U	E-166	R	S	1.6
1501	A	G	E-179	K	E	4.9
1701	A	C	E-245	P	P	1.8
1795	A	G	E-277	N	D	1.3
1804	A	G	E-280	K	E	1.9
1804	A	C	E-280	K	Q	2.6
1826	A	G	E-287	K	R	1.6
1832	G	A	E-289	R	K	1.3
1894	A	C	E-310	K	Q	1.9
2066	C	U	E-367	A	V	2.7
2229	A	G	E-421	L	L	1.2
7977	A	G	NS5-99	E	E	1.1
8119	A	C	NS5-147	I	L	1.1
8849	A	G	NS5-390	E	G	2.2
9934	A	U	NS5-752	N	Y	1.6
10906	A	G	3’ UTR	–	–	1.4

## Discussion

Based on robust literature describing the important role of the E-E138K mutation for the attenuation of the JEV live attenuated vaccine SA14-14-2, we hypothesized that the homologous mutation in WNV would confer an attenuated phenotype. The genome of the rescued WNV E-E138K mutant had one additional mutation at nucleotide 1077, but as this mutation does not encode an amino acid substitution, we hypothesized that it would not significantly modify the virulence phenotype. In vitro characterization of the WNV E-E138K mutant suggested that the virus had a similar phenotype to the parental NY99ic. Specifically, TS assays, multiplication kinetics, and 36 hpi cytokine quantification did not identify strongly distinct phenotypic differences between the E-E138K mutant and the parental NY99ic. It is possible that the E-E138K mutant may decrease inflammation based on the attenuated production of IL-6 compared with NY99ic. Although changes in inflammatory cytokines (including IL-6) have been linked to the E protein in other studies,^16^ it is not clear whether the difference of ~1 pg/mL observed in this study would have biological relevance in humans or other hosts.

To test whether the E-E138K mutation would confer attenuation in WNV, outbred Swiss Webster mice were utilized as a model, as they are highly susceptible to WNV, and the i.p. LD_50_ for the parental NY99ic is between 0.1 and 10 PFU.^17–20^ The E-E138K mutation was shown to attenuate JEV for both mouse neuroinvasion and neurovirulence in previous studies. For example, the E-E138K mutation in a JEVic was attenuated 1000-fold from the parental virus for both neuroinvasion and neurovirulence in the BALB/c mouse model.^9^ Furthermore, two studies of different JEV strains harboring the E-E138K mutation reported that the mutation conferred complete attenuation of neuroinvasion in inbred immunocompetent mice inoculated with either 100,000 PFU or 50 million PFU, whereas the parental strains were completely lethal.^21,22^ Although there is robust literature studying the mouse attenuation phenotype of the E-E138K mutation in JEV, fewer studies have been completed in WNV. WNV New York 1999-flamingo 382-99 strain serial passage in human adrenal gland epithelial SW13 cells resulted in the E-E138K mutation and this was mildly attenuating 50-fold by the intracranial (i.c.) route and 200-fold by the i.p. route; however, it is not known whether this virus had compensatory mutations in the NS genes that contributed to the virulence phenotype.^12^ Similarly, the E-E138K mutation decreased neurovirulence of a chimeric WNV vaccine candidate, Chimerivax-WNV, which combines the structural genes of WNV NY99 with the NS genes of the YFV 17D vaccine.^15^ An infectious DNA (iDNA) vaccine platform based on the lineage 2 WNV isolate W956 with an engineered E-E138K mutation was not lethal in outbred mice when administered by intramuscular or intradermal routes with either 1 μg or 100 ng of iDNA, but the mutant iDNA retained virulence when inoculated by the i.c. route.^13^ Based on the potential for E-E138K to confer mouse attenuation in JEV and WNV, it was anticipated that the WNV NY99ic E-E138K mutant would have at least partial attenuation of neuroinvasion; therefore, both a relatively low dose (500 PFU) and a high dose (82 million PFU) were tested by the i.p. route. Although 500 PFU of NY99ic is lethal in outbred mice, this was chosen as a low dose based on the strong attenuation of neuroinvasion that the JEV E-E138K mutant conferred in outbred mice (i.p. LD_50_ > 10^5^ PFU).^12^ Surprisingly, all mice succumbed to infection by the i.p. route; therefore, the virus was not administered by the i.c. route. It is possible that the WNV E-E138K mutant is very mildly attenuated as demonstrated for SW13 cell-passaged WNV,^12^ but low-level attenuation would not be ideal for live attenuated vaccine development; thus, lower doses were not evaluated.

One explanation for the virulence of the WNV E-E138K mutant is that the mutation is not stable. Multiple studies have reported that E-E138K is a stable mutation when JEV is passaged in cell culture;^8,11,23^ however, in JEV and in the WNV iDNA platform, the mutation was capable of reversion after passage in mouse brain.^8,9,14^ Previous studies utilized Sanger sequencing that provides only a consensus sequence, whereas NGS allows for a more detailed analysis of nucleotide diversity. Measurement of nucleotide diversity with Shannon entropy and measurement of significant SNVs revealed that the WNV E-E138K mutant had a high frequency of mutations concentrated in the region of the E-protein gene. Although the E-E138K mutation was stable in the consensus sequence, there was evidence of a relatively large (14.2%) SNV encoding reversion of K138E in the unpassaged virus. Due to the similarity between glutamic acid (GAA/GAG) and lysine (AAA/AAG), only the first nucleotide in the codon can be changed to induce the desired mutation at E-138. Thus, mutation of the first nucleotide (and not the third variable base) suggests that the virus is undergoing selection for the revertant subpopulation. The E-E138K mutation is associated with cell culture adaptation of glycosaminoglycan (GAG) binding in both JEV and WNV;^9,12,21^ therefore, it is not surprising that reversion of E-K138E was not evident in the consensus sequences of Vero-passaged P1 and P5 stocks of the mutant. Both the P1 and P5 stocks of the E-E138K mutant did show evidence of reversion in the SNVs, but as these SNVs were significantly strand biased, we cannot be confident that they represent true variants. Importantly, the evidence of reversion in the SNVs of the unpassaged P0 stock of the WNV E-E138K mutant demonstrates that the virus is capable of rapidly selecting for the parental amino acid. In addition, the P1 virus was used for the mouse studies and was found to revert in vivo regardless of the absence of reversion (without strand bias) in the cell culture stock.

**Fig. 5:**
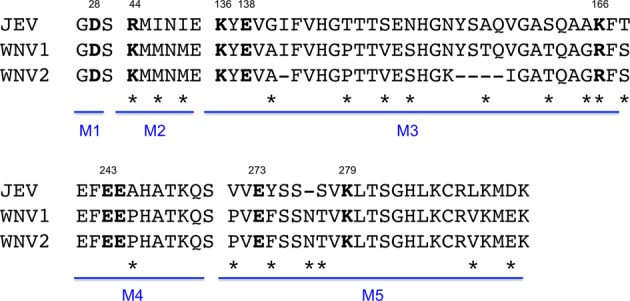
Alignment of WNV and JEV structural motifs reveals amino acid differences between the viruses. Amino acid sequences of JEV strain SA14 (accession number ANV81277.1), WNV lineage 1 (WNV1) strain NY99 (accession number AAF20092.2), and WNV lineage 2 (WNV2) strain W956 (accession number NP_041724.2) were aligned. The five motifs responsible for the hole between envelope protein dimers are denoted as M1–M5. Amino acids in bold are the nine charged residues that directly cause the hole and amino acids that are denoted with an asterisk are residues that are different between WNV1 (used in this study) and JEV.

Although there has been evidence of E-K138E reversion in JEV, it is a relatively infrequent outcome as the mutation typically remains stable and induces attenuation of JEV mouse neuroinvasion and neurovirulence.^7,8^ Insights into the location of E-E138K on the structure of the WNV E protein compared with that of the JEV E protein may help to explain why the mutation is poorly tolerated in WNV and does not attenuate the virus. Structural studies of JEV demonstrated that when the E monomers form a dimer on the viral surface, the dimer has unique “holes” between monomers that are specific to the encephalitic flaviviruses, whereas hemorrhagic flaviviruses such as DENV have more interactions between monomers and thus have less prominent holes.^21^ The study postulates that the E dimer hole is functionally important for virus entry into the nervous system.^21^ E-138 is one of nine electrostatic residues responsible for this unique hole along with D28, R44, K136, K166, E243, E244, E273, and K279.^21^ The encephalitic hole in JEV is the result of 5 amino acid motifs responsible for the positioning of the 9 electrostatic amino acids listed above, and JEV and WNV differ at 19 of 77 amino acids within the 5 motifs^21^ (Fig. 5). Therefore, the position of E-E138 in WNV may not be identical to the position in JEV, ultimately allowing mutation of this electrostatic residue to be better tolerated in JEV than in WNV. Interestingly, lineage 1 and 2 WNVs have identical amino acids in the five motifs with the exception of motif 3 (Fig. 5). Motif 3 harbors electrostatic E residues 136, 138, and 166, and therefore the differences in attenuation between WNV lineage 1 and 2 could in part explain why the lineage 2 virus used in other studies can more stably harbor the E-E138K mutation.^13,14^ The NGS data showed that mutation of E-E138K in WNV not only caused reversion of K138E in an SNV, but five other SNVs are prevalent in structurally neighboring residues, including K136Q, R166T, R166S, K280E, and K280Q (K280 in WNV is homologous to K279 in JEV) (Table 3). Thus, three of the nine electrostatic residues responsible for the hole in the JEV E protein have evidence of mutation when E-E138K is mutated in WNV. Each of the SNVs surrounding the encephalitic hole decreases the positive charge of the local environment, suggesting that WNV is increasing mutation near E-138 to try to compensate for the electrostatic charge change that occurs with mutation of E-E138K. Investigation of E-E138K in the WNV iDNA vaccine platform found that loss of positive charge at E-166 by mutation of R166I or R166S stabilized the gain of positive charge resulting from the E-E138K mutation and thus prevented reversion to virulence at E-138.^14^ Notably, each of the five SNVs that structurally neighbor E-138 are associated with a loss of positive charge by mutation of either lysine or arginine, supporting the hypothesis that the virus is trying to compensate for the lysine at E-138. It is possible that fixation of an amino acid that is not positively charged at E-136, E-166, or E-280 would stabilize the E-E138K mutation and prevent reversion to virulence.

In summary, the E-E138K mutation does not confer attenuation in WNV even though it is strongly attenuating for mouse neuroinvasion and neurovirulence of JEV. In JEV, it has been demonstrated that E-E138K will infrequently revert if the virus is passaged in mouse brain; however, the mutation is stable when passaged in cell culture.^8,9^ This report has found that E-E138K is not stable in WNV and begins to revert upon rescue of the transfected virus prior to passage. The E-E138K mutation was considered for the candidate chimeric WNV/YFV 17D vaccine, ChimeriVax-WN02. The first generation of this vaccine platform, ChimeriVax-WN01, retained low-level neurovirulence in mice and was associated with several severe adverse events after its licensure for veterinary use.^15,24^ To improve the safety of ChimeriVax-WNV, it was proposed to include several JEV SA14-14-2 E-protein mutations that are important for attenuation of mouse neurovirulence and presumably human virulence.^15^ Although E-E138K did decrease mouse neurovirulence of ChimeriVax-WNV, the mutations E-L107F, E-A316V, and E-K440R were ultimately selected for further development of ChimeriVax-WN02, as they caused more profound neurovirulence attenuation than E-E138K in outbred ICR mice.^15^ Our data demonstrating that E-E138K mutation is not stable in WNV is consistent with this study that the residues E-107, E-316, and E-440 would be better targets for mutagenesis of ChimeriVax-WN02, as these residues are not structurally neighboring E-138. Although E-E138K is an important determinant of attenuation for the live vaccine JE SA14-14-2, the equivalent mutation in WNV does not confer significant attenuation, but instead it increases variation of the viral E protein and would not be a safe mutation to include in a candidate WNV vaccine.

## Methods

### Cell Culture

Vero African Green Monkey kidney cells and A549 human alveolar epithelial cells were grown at 37 °C with 5% CO_2_ in minimum essential media (MEM) supplemented with 100 U/mL penicillin, 100 μg/mL streptomycin, 2 mM l-glutamine, 0.1 mM non-essential amino acids, and 8% fetal bovine serum (FBS).

### Generation of WNV Infectious Clones

Viruses were generated using a WNV infectious clone based on strain NY99-flamingo 382-99 (referred to as NY99ic).^17,18,20,25,26^ The NY99ic comprised two plasmids, one containing the 5′-untranslated region (UTR) and the virus structural genes, and a second containing the NS genes through the 3′-UTR. Quikchange II XL Site-Directed Mutagenesis Kit (Agilent) was utilized to generate the WNV E-E138K mutant using the 5′-plasmid. The mutagenesis primers were 5′-CATGGACAAAAATGGCCACCTTGTACTTGATATTCTCTTTCAAG-3′ and 5′-CTTGAAAGAGAATATCAAGTACAAGGTGGCCATTTTTGTCCATG-3′. After mutagenesis, plasmids were transformed into MC1061 competent *Escherichia coli* cells and grown in 200 mL Luria broth with 100 μg/mL ampicillin. After growth for 14–16 h, bacteria were pelleted and suspended in glucose-tris-EDTA buffer. Cells were lysed using 0.2 M NaOH/1% SDS and lysis was neutralized using 3 M KOAc. After isopropanol precipitation, the plasmid was treated with RNAse A for 60 min, then purified using phenol:chloroform:isoamyl alcohol. The purified plasmid was ethanol precipitated, then desalted and concentrated using the QiaQuick PCR purification kit (Qiagen). Three micrograms of the 5′-plasmid and 6 μg of the 3′- plasmid were digested for 2 h at 37 °C with NgoMIV and XbaI, and then plasmids were ligated by incubating with T4 DNA ligase at 4 °C overnight. The ligase was inactivated at 70 °C for 10 min and then linearized with XbaI for 2 h at 37 °C. Phenol:chloroform extraction was utilized to purify the linearized plasmid and the DNA was precipitated at −20 °C overnight using ethanol and 3 M pH 5.2 NaOAc. After drying the purified DNA, in vitro transcription was completed using the Ampliscribe T7 High Yield Transcription Kit (Lucigen). After ~3 h, the transcription reaction was added to 3.4 × 10^6^ Vero cells in 500 μL phosphate-buffered saline (PBS) and electroporated with two pulses at 1.5 kV, 25 μF, ∞ ohms using a Gene Pulser electroporator (Bio-Rad). Cells were grown in MEM media (prepared as described above) at 37 °C with 5% CO_2_ until cytopathic effect became apparent. Viruses were rescued in Vero cells between 3 and 5 days post transfection, and stocks were stored at −80 °C. Viruses were passaged once in Vero cells to generate the stocks utilized for in vitro and in vivo experiments described below.

### Temperature Sensitivity Assays

Infectivity titers of viruses rescued by reverse genetics were determined in duplicate with plaque assays at both 37 °C and 41 °C. Ten-fold serial dilutions of viruses (10^−1^−10^−6^) were added to six-well plates of Vero cells and incubated at room temperature for 30 min. Cells were overlaid with MEM media containing 1% agar and incubated at 37 °C or 41 °C with 5% CO_2_. After 2 days, a second overlay was added containing 2% neutral red dye. Plaques were counted on days 3 and 4 post infection.

### Multiplication Kinetics

Duplicate flasks of Vero cells and A549 cells were infected with a multiplicity of infection (MOI) of 0.1. After incubating the virus with the cells for 30 min at room temperature, MEM containing 2% FBS was added to the cells and the flasks were incubated at 37 °C with 5% CO_2_ for 4 days. At 0, 24, 36, 48, 72, and 96 hpi, 2 aliquots were collected from each flask, centrifuged at 367 × *g* for 5 min, and supernatants were stored at −80 °C until titration for infectivity using plaque assays as described above.

### Quantification of Cytokines

A549 cells grown in six-well plates were infected with an MOI of 0.1 of either NY99ic, the E-E138K mutant, or PBS, which was used as a mock infection. At 36 hpi, supernatants were collected from each well as described for multiplication kinetics experiments above. Samples of each supernatant were gamma-irradiated using 5 mrad to remove infectivity and cytokines were then measured using the Bio-Plex Pro Human Cytokine 27-Plex Assay (Bio-Rad) and a Bio-Plex custom assay for human IFN-α2 and IFN-β. Bio-Plex assays were performed according to the manufacturer’s guidelines. Cytokine levels of NY99ic were compared with the E-E138K mutant and with mock-infected cells by using a Kruskal–Wallis test with Dunn’s multiple comparisons.

### Mouse Infection

Groups of 4-week-old female NIH Swiss Webster outbred mice (Taconic Farms, Germantown, NY) were utilized to evaluate attenuation of neuroinvasion. Mice were inoculated by the i.p. route with an inoculum of 500 PFU and an additional group of mice was also inoculated i.p. with 8.2 million PFU of the E-E138K mutant. All animal experiments complied with the National Institutes of Health guide for the care and use of laboratory animals and were approved by the Institutional Animal Care and Use Committee of the University of Texas Medical Branch.

### Isolation of Virus from Mouse Brain

Brains were collected from five mice that succumbed to a 500 PFU inoculum of the E-E138K mutant. The brains were frozen at −80 °C until homogenization. Approximately half of each brain was homogenized with 30 cycles/second for 2 min in 500 μL of MEM with 2% FBS using the Qiagen TissueLyser II. Homogenates were immediately placed on ice prior to centrifugation at 4 °C at a speed of 9184 × *g* for 10 min. The supernatants were collected and immediately titrated using plaque assays prior to storage at −80 °C.

### Nucleotide Sequencing Analysis

RNA was extracted from Vero cell culture supernatant or from mouse brain homogenate using the QiaAmp Viral RNA Kit (Qiagen). RNA from mouse brain homogenate was amplified using PCR primers specific to the E protein and PCR products were purified using the QiaQuick PCR Purification Kit (Qiagen) prior to Sanger sequencing. For NGS of cell culture-derived viral RNA, paired-end reads were sequenced on the Illumina NextSeq 550 platform. Trimmomatic^27^ was utilized to remove adapters and any sequences with a quality score below 30. The trimmed reads were aligned to a NY99ic reference sequence using Bowtie2 with the very sensitive local parameter. All reads were sorted based on genome position and coordinate position using SAMtools v. 1.3 and PCR duplicates were marked and removed using Picard Tools v. 1.119 (Broad Institute) with the optical duplicate pixel distance set to 0. Depth of coverage was determined using SAMtools. V-Phaser 2 (Broad Institute) was utilized to measure significant SNVs in the viral RNA populations.^28^ To measure the absolute diversity at each nucleotide position, Shannon entropy was calculated^29,30^ using the formula $Sn = - \frac{{\mathop {\sum }\nolimits_{i = 1}^n fi\left( {lnfi} \right)}}{N}$, where *n* is the number of possible nucleotides identified, *fi* is the observed frequency of a variant, and *N* is the total number of clones analyzed. Mean genomic entropy for NY99ic and the E-E138K mutant were compared using a Mann–Whitney test.

### Statistical Analysis

Kruskal–Wallis tests with Dunn’s post-hoc correction, Mann–Whitney tests, and measurements of SD were each performed using GraphPad Prism version 8.0.

### Reporting Summary

Further information on experimental design is available in the Nature Research Reporting Summary linked to this paper.

## Supplementary information

Supplementary Table 1

Reporting Summary

## Data Availability

All unique biological materials and the corresponding datasets generated and analyzed during the current study are available from the corresponding author on reasonable request.
