# Supplementary material for: Japanese encephalitis vaccine-specific envelope protein E138K mutation does not attenuate virulence of West Nile virus
Source: NPJ Vaccines. 2019 Dec 5;4:50. doi: 10.1038/s41541-019-0146-0 (PMC6895119; doi:10.1038/s41541-019-0146-0)
Supplement: Supplementary file 1 — Supplementary Table 1 [file 41541_2019_146_MOESM1_ESM.pdf]

Supplementary Table 1: Cytokine detection at 36 hpi in A549 cell culture supernatant of NY99ic and E-E138K mutant infected cells

Not detected	No change	Statistical Change
IL-1β	IFN-γ	IL-6
IL-12p70	TNF-α	
IL-1RA	IFN-α2	
IL-10	IFN-β	
IL-15	IL-4	
IL-17	IL-5	
PDGF	IL-9	
	IL-13	
	IL-2	
	IL-7	
	FGF	
	VEGF	
	G-CSF	
	GM-CSF	
	CXCL8/IL-8	
	CCL11/Eotaxin	
	CXCL10/IP-10	
	CCL2/MCP-1	
	CCL3/MIP-1α	
	CCL4/MIP-1β	
	CCL5/RANTES	

Low detection refers to cytokines that were detected in quantities < 1 pg/mL and were not detected in all replicates tested. Significance comparing the mutants and mock to NY99ic was tested using a Kruskal-Wallis test with Dunn's multiple comparisons.
IL = interleukin, IFN = interferon, TNF = tumor necrosis factor, PDGF = platelet-derived growth factor, FGF = fibroblast growth factor, VEGF = vascular endothelial growth factor, GM-CSF = granulocyte-macrophage colony-stimulating factor, G-CSF = granulocyte colony-stimulating factor, CXCL and CCL = chemokine ligand, MIP = macrophage inflammatory protein, IP = interferon gamma-induced protein, MCP = monocyte chemoattractant protein, RANTES = regulated on activation, normal T cell expressed and secreted
